# Decoding Immune Dysregulation in Sepsis Through Extracellular Vesicles: A Path to Precision Medicine

**DOI:** 10.3390/pharmaceutics18050570

**Published:** 2026-05-04

**Authors:** Martina Schiavello, Barbara Vizio, Ornella Bosco, Chiara Dini, Emanuele Pivetta, Fulvio Morello, Enrico Lupia, on behalf of the SEPSIS in Emergency Medicine Study Group

**Affiliations:** 1 Department of Medical Sciences, University of Turin, 10126 Turin, Italy; martina.schiavello@unito.it (M.S.); barbara.vizio@unito.it (B.V.); ornella.bosco@unito.it (O.B.); chiara.dini@unito.it (C.D.); emanuele.pivetta@unito.it (E.P.); fulvio.morello@unito.it (F.M.); 2 Residency Program in Emergency Medicine, University of Turin, 10126 Turin, Italy

**Keywords:** sepsis, immune dysregulation, extracellular vesicles

## Abstract

Sepsis remains a leading cause of mortality worldwide and is increasingly recognized as a syndrome of dynamic immune dysregulation rather than a uniform inflammatory condition. The traditional paradigm of sequential hyperinflammation followed by immunosuppression has been replaced by a more complex view in which these processes coexist and evolve over time, contributing to marked interindividual variability in clinical outcomes. Despite advances in supportive care, current diagnostic and therapeutic approaches are still largely non-specific and fail to account for this biological heterogeneity. Extracellular vesicles (EVs) have emerged as key mediators of intercellular communication and potential integrators of immune activity in sepsis. These nanosized particles carry proteins, nucleic acids, lipids, and metabolites that reflect the functional state of their cells of origin and actively participate in immune regulation. Experimental and clinical evidence indicate that EVs exert context-dependent effects, contributing both to the propagation of inflammatory processes and the establishment of immunosuppressive states through the transfer of regulatory signals. Beyond their mechanistic role, EVs represent a promising platform for immune monitoring. Their cell-specific and dynamic molecular signatures have been associated with disease severity, organ dysfunction, and clinical trajectories, suggesting their role as biomarkers for patient stratification. In parallel, engineered and stem cell-derived EVs are being explored as therapeutic vectors capable of modulating immune responses and restoring immune homeostasis. In this review, we examine current concepts of immune dysregulation in sepsis and discuss how EVs may serve as both mediators and decoders of immune heterogeneity. We propose that EV-based approaches could bridge the gap between high-dimensional immunological profiling and precision immunotherapy, enabling more adaptive and individualized management of septic patients.

## 1. Introduction

Sepsis is widely recognized as one of the most complex medical challenges in modern healthcare [1]. The concept of sepsis and its definition have substantially evolved over time. Historically used to describe a state of putrefaction associated with infection [2], the term has gradually acquired a more precise biological meaning [2]. According to the current consensus definition, Sepsis-3, sepsis is defined as a life-threatening organ dysfunction caused by a dysregulated host response to infection [3]. Within this framework, the notion of *dysregulated host response* encompasses maladaptive processes involving both immune and non-immune pathways that ultimately contribute to tissue injury, organ dysfunction, and death [1,4,5].

Recent estimates from the Global Burden of Diseases study underscore the magnitude of the problem, indicating that sepsis remains a major global health burden, accounting for an estimated 50 million incident cases and approximately 11 million deaths annually worldwide [6,7,8]. When patients with sepsis require admission to critical care units, one in three patients do not survive 30 days [9,10] and mortality varies by age, comorbid status, number, and type of organ dysfunction [11,12].

Although advances in critical care have reduced case fatality rates over the past few decades, the overall incidence of sepsis continues to rise, largely driven by population aging, expanded use of invasive procedures, and widespread exposure to immunosuppressive therapies [6]. Beyond being a major cause of morbidity and mortality among hospitalized patients, sepsis’s increasing incidence is also partly linked to advances in medical care that allow the survival of individuals with severe underlying diseases who are consequently more susceptible to infection [13]. 

These trends highlight the limitations of current treatments, which are predominantly supportive and include measures such as source control, timely antibiotics, resuscitation, and supportive care for organ dysfunction [14,15].

Despite extensive preclinical and clinical investigation, immunomodulatory and immunoadjuvant therapies have failed to demonstrate consistent benefits in randomized trials [16,17,18]. A major contributor to these failures is the lack of patient stratification by immune status, leading to the indiscriminate application of targeted interventions across biologically distinct patient populations [19]. 

In parallel with the progressive understanding of sepsis pathophysiology, the classical view of sepsis as a biphasic condition—characterized by an initial hyperinflammatory phase followed by a compensatory immunosuppressive state—has been progressively revised [5]. Hyperinflammation and immunosuppression are now recognized as overlapping and dynamically evolving processes, the relative dominance of which varies over time and across individuals [5,20,21]. Among these alterations, sepsis-associated immunosuppression has emerged as a key determinant of secondary infections, prolonged intensive care unit stay, and late mortality [17]. However, clinical and laboratory tools capable of capturing immune dysfunction in real-time and monitoring its evolution have yet to be fully established. Widely used biomarkers, including C-reactive protein, interleukin-6, and procalcitonin, primarily reflect systemic inflammation and provide only partial insight into immune function [17]. 

Extracellular vesicles (EVs) are membrane-bound particles released by virtually all cell types that transport diverse molecular cargo—including proteins, nucleic acids, lipids and metabolites—reflecting the activation state and functional phenotype of their cells of origin [22,23]. By mediating intercellular communication, EVs participate actively in immune regulation and may provide a dynamic readout of immune dysregulation [24,25,26]. Emerging evidence suggests that EVs may function both as contributors to sepsis pathophysiology and as accessible biomarkers with translational relevance [27,28,29]. The ability to integrate mechanistic insight with measurable biological signals positions EVs at the interface between immune monitoring and therapeutic decision-making [30].

Here, we aim to review the current knowledge on immune dysregulation in sepsis, with particular emphasis on the mechanisms that reveal the simultaneous emergence of inflammatory and immunosuppressive mechanisms, and to examine how these processes contribute to the remarkable immune heterogeneity in sepsis. We particularly focus on EVs, which have emerged as both mediators of intercellular communication and biomarkers of immune status. By integrating mechanistic insights with advances in high-dimensional analytical approaches, EVs may offer new opportunities to capture dynamic immune trajectories and support patient stratification in trials of targeted immunomodulatory therapies. 

To this end, although this review is structured as a narrative overview and does not adhere to a formal systematic methodology, we identified the relevant literature through targeted searches on PubMed using combinations of keywords such as “sepsis”, “immune dysregulation” and “extracellular vesicles”, and selected the most pertinent studies based on their relevance to the topic, with emphasis placed on recent experimental, translational, and clinical evidence.

## 2. Immune Dysregulation in Sepsis

### 2.1. When Defense Becomes Harm: The Dual Faces of the Immune Response in Sepsis

Sepsis is associated with substantial short- and long-term mortality [3]. Although many patients survive their initial infectious insult, a significant proportion experience prolonged intensive care unit (ICU) stays complicated by secondary infections, persistent organ dysfunction, and impaired immune competence—commonly described as sepsis-induced immunosuppression [5,31].

The immune response to infection is intrinsically protective [20,32]. Under physiological conditions, coordinated pro- and anti-inflammatory mechanisms promote pathogen clearance while maintaining tissue homeostasis [33]. Pathogen-associated molecular patterns (PAMPs) are recognized by pattern recognition receptors (PRRs), including Toll-like receptors (TLRs), expressed on innate immune cells, triggering inflammatory mediators and the activation of antimicrobial pathways [20,33].

However, in sepsis, this tightly regulated system becomes destabilized [34]. Failure to eradicate the pathogen or excessive stimulation of innate immune pathways leads to a maladaptive response characterized by excessive inflammation, profound immune suppression, or—more commonly—a coexistence of both states [5,35]. Hyperinflammation may culminate in endothelial dysfunction, microvascular thrombosis, and organ failure, whereas sustained immunosuppression impairs pathogen clearance and increases the susceptibility to secondary infections and viral reactivation [4,5,32]. 

These systemic alterations are reflected by functional changes in key immune cell populations [20]. For instance, monocytes and macrophages from septic patients frequently display reduced cytokine production following ex vivo stimulation—a phenomenon commonly referred to as *immunoparalysis* [4,20]. This is associated with decreased expression of major histocompatibility complex (MHC) class II molecules (notably reduced monocyte HLA-DR), impaired antigen-presenting capacity, and transcriptional reprogramming of innate immune cells [4,20]. At the same time, other adaptive immune alterations, which include lymphocyte apoptosis, expansion of regulatory T cells, and features of T-cell exhaustion, such as increased expression of inhibitory receptors, including programmed death-ligand 1 (PD-1), develop and participate in these functional changes [4,36]. Together, these phenomena reflect a coordinated and multi-layered suppression of immune function rather than an isolated cellular defect [36]. This functional reprogramming represents one manifestation of the broader immune dysregulation that characterizes sepsis and contributes to the increased vulnerability to secondary infections observed during the later stages of the disease [4]. Together, these findings indicate that immune dysregulation is not merely a consequence of sepsis but a central determinant of disease trajectory and outcome [34,37,38,39]. 

### 2.2. Phases of Immune Dysregulation in Sepsis: Hyperinflammation, Immunosuppression and Beyond

The dysregulated host response in sepsis reflects a complex interplay of soluble mediators, cellular activation states, metabolic shifts, and transcriptional reprogramming that collectively influence patients’ clinical trajectories [36,38]. Rather than representing a uniform biological process, sepsis encompasses heterogeneous and dynamically evolving immune responses that vary substantially among patients and over time [17]. In the past, it was assumed that all sepsis cases start as a hyperinflammatory response to the infectious insult, followed by a hypoinflammatory response, and that patients died from immunosuppression [13,19,31]. Over the last decade, accumulating experimental and clinical evidence indicates that, at the onset of the disease, the immune response greatly varies from hyperinflammation to hypoinflammation [5]. This generates the need to classify patients early for immune function [40]. Sepsis is therefore better described in terms of a patient-specific immune trajectory in which inflammatory and suppressive pathways coexist and fluctuate over time rather than presenting in discrete sequential phases [5,19,40].

#### 2.2.1. Hyperinflammation

The hyperinflammatory phase of sepsis is initiated by the recognition of pathogen-associated molecular patterns (PAMPs) and damage-associated molecular patterns (DAMPs) by pattern recognition receptors (PRRs) expressed on circulating and tissue-resident immune cells [41]. Activation of these pathways triggers a rapid and coordinated host response, characterized by cytokine release, leukocyte recruitment, complement activation, and amplification of inflammatory signaling cascades [34,36,41]. 

While these mechanisms are essential for effective pathogen clearance, their dysregulated activation can lead to excessive systemic inflammation. This state is associated with endothelial activation, increased vascular permeability, and microvascular thrombosis, ultimately contributing to organ dysfunction [42,43,44,45]. In this setting, the so-called *cytokine storm* represents the clinical and biological manifestation of uncontrolled inflammatory amplification, reflecting not only the release in the circulation of elevated concentrations of pro-inflammatory mediators—such as IL-6, TNF, and IL-1β—but also widespread cellular activation and tissue injury [42,43].

From a clinical perspective, biomarkers including C-reactive protein (CRP), procalcitonin (PCT), and IL-6 have been widely used as surrogates of inflammatory burden [43]. However, these markers primarily capture systemic inflammation and provide limited insight into immune cell function or patient-specific immune trajectories [4]. Consistent with this limitation, therapeutic strategies targeting individual inflammatory mediators—such as anti-TNF agents or IL-1 receptor antagonists—have repeatedly failed to demonstrate a survival benefit in unselected septic populations [16,31]. These findings highlight the inadequacy of interpretative models centered on inflammation to fully capture the complexity of the host immune response in sepsis.

#### 2.2.2. Immunosuppression

In parallel with inflammatory activation, sepsis is characterized by a profound and often persistent state of immunosuppression [21,36,40]. This condition extends beyond a simple compensatory anti-inflammatory response and rather reflects a coordinated reprogramming of both innate and adaptive immune compartments [21,46].

At the cellular level, sepsis-induced immunosuppression is associated with widespread lymphocyte apoptosis, functional impairment of T cells (consistent with exhaustion), expansion of regulatory T cells, accumulation of myeloid-derived suppressor cells, and reduced expression of the MHC class II molecules (HLA-DR) on monocytes—most notably decreased HLA-DR expression—indicating impaired antigen presentation capacity [5,20,47]. These alterations vary considerably in magnitude and duration across patients, contributing to the marked immunological heterogeneity observed in sepsis.

This immunosuppressive state is not static. Immune function evolves over time, with individual patients following distinct immune trajectories rather than exhibiting a fixed phenotype [40]. Moreover, immune alterations may also be compartmentalized, as circulating immune cells only partially reflect tissue-level responses, further complicating the assessment of immune status in clinical settings [36].

Despite growing recognition of these mechanisms, their translation into effective therapeutic strategies has been limited [38,40,48]. Interventions designed either to suppress excessive inflammation or to restore immune function—such as cytokine blockade or immunostimulatory therapies—have produced inconsistent results in clinical trials [4,31]. A central limitation has been represented by the lack of reliable tools to stratify patients according to immune status and to guide treatment in a dynamic, individualized manner [40]. These considerations highlight the need for biomarkers capable of capturing immune function in a dynamic and cell-specific way.

#### 2.2.3. Beyond the Dichotomy: Immune Reprogramming and Persistent Inflammation—Immunosuppression and Catabolism Syndrome (PICS)

Sepsis is increasingly viewed not as a sequence of alternating hyperinflammatory and immunosuppressive phases, but as a syndrome of immune reprogramming [36,37,38,49]. This perspective reflects the recognition that host responses are sustained, adaptive, and context-dependent, rather than transient and self-limited [2,50]. A subset of patients who survive the initial insult develops a distinct clinical phenotype characterized by persistent low-grade inflammation, immune dysfunction, ongoing organ injury, and progressive catabolism, termed persistent inflammation—immunosuppression and catabolism syndrome (PICS) [49,51]. This condition represents a maladaptive state of sustained immune and metabolic reprogramming, and it is associated with delayed recovery, increased susceptibility to secondary infections, and poor long-term outcomes [51]. 

At the biological level, this state is maintained by coordinated alterations across multiple pathways, including sustained myeloid activation, expansion of myeloid-derived suppressor cells, mitochondrial dysfunction, metabolic rewiring, and epigenetic remodeling of immune cells [49]. These processes indicate that hyperinflammation and immunosuppression are not opposing phases, but interconnected features of a broader remodeling of the immune system that affects both host defense and tissue homeostasis [51].

Currently available biomarkers capture only limited aspects of this complexity and do not provide an integrated and/or dynamic assessment of immune function at the cellular level [50]. As a result, our ability to monitor patients’ immune trajectories longitudinally and in a cell-specific manner at the bedside is limited, representing a major barrier to the development of precision immunomodulatory strategies [16,31]. 

These limitations have driven growing interest in biological systems capable of integrating signals across multiple cellular compartments. Among these, EVs have emerged as promising candidates, given their ability to reflect and modulate immune processes in a dynamic and cell-specific manner.

## 3. Extracellular Vesicles as Mediators of Immune Dysregulation in Sepsis

EVs constitute a highly heterogeneous population, encompassing exosomes, microvesicles, and apoptotic bodies, with partially overlapping biogenesis pathways and size distributions. In line with MISEV2023 guidelines, standardization of EV isolation, characterization, and nomenclature remains essential to ensure reproducibility and comparability across studies [52]. This heterogeneity has important implications for data interpretation, particularly when comparing findings across experimental and clinical settings [53]. Against this background, EVs are increasingly recognized as key mediators of intercellular communication in complex inflammatory diseases [52]. Rather than being passive cellular by-products, EVs function as structured carriers of biological information, encapsulating proteins, nucleic acids, lipids, and metabolites that reflect the activation state and functional phenotype of their cells of origin [23].

Advances in analytical technologies have markedly improved the characterization of EV populations [54,55]. Approaches such as single-vesicle analysis, high-dimensional profiling, and machine learning-based clustering are beginning to uncover EV-derived *immune fingerprints* associated with clinical trajectories, organ dysfunction, and treatment responses in sepsis [56,57,58]. These approaches move beyond simple quantification and enable the identification of functionally distinct EV subpopulations, supporting the concept of dynamic immune phenotyping [59,60].

In sepsis, EVs provide a unique window into ongoing immune processes. Their molecular cargo changes in response to inflammatory stimuli, metabolic stress, and immune exhaustion, effectively revealing the functional state of their parent cells [26,28,61,62,63,64]. In addition, through the transfer of bioactive molecules, EVs may influence multiple aspects of disease pathophysiology, including immune activation, coagulation pathways, endothelial function, and tissue injury [26,62,63].

Therefore, EVs exhibit a dual biological role. They act as reporters of immune status, reflecting cell-specific responses during infection [26,56], while simultaneously contributing to the propagation and modulation of host responses through the transfer of regulatory signals between cells [26]. By capturing dynamic and cell-specific alterations, EVs offer a powerful tool useful for investigating immune trajectories in sepsis. This perspective aligns with the growing recognition of sepsis endotypes identified through transcriptomic and immune profiling approaches, which aim to define biologically distinct patient subgroups with divergent clinical outcomes and treatment responses [53,65]. In this context, EV-based profiling may provide a complementary approach by enabling longitudinal and cell-resolved insights into immune activity. By reflecting ongoing intercellular communication and functional immune states, EVs could refine current endotyping frameworks. Examining EV populations according to their cellular origin further enhances this framework, as vesicles derived from different cell types exert distinct and sometimes even opposing biological effects. In addition, since most of the data currently available refer to single-time-point assessments, which potentially do not fully reflect the temporal evolution of immune responses in sepsis, EV profiling may help in supporting a more temporally resolved stratification of septic patients. 

**Figure 1:**
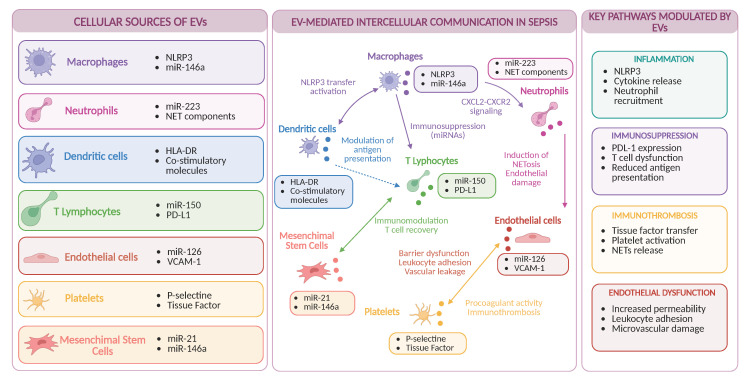
Extracellular vesicles as mediators of immune dysregulation in sepsis.

A schematic overview of the role of EVs in sepsis-associated immune dysregulation is shown in Figure 1.

Extracellular vesicles (EVs) released from immune (macrophages, neutrophils, dendritic cells, and T lymphocytes) and non-immune cells (endothelial cells, platelets, and mesenchymal stem cells) carry bioactive cargo, including protein and regulatory microRNAs, reflecting the activation state of their cells of origin. Through the transfer of specific molecular signals (e.g., NLRP3, CXCL2, PD-L1, tissue factor, and miR-146a), EVs mediate intercellular communication and modulate key pathways involved in sepsis, including inflammation, immunosuppression, immunothrombosis, and endothelial dysfunction. The functional impact of EVs varies according to their cellular source, with vesicles derived from innate immune cells predominantly amplifying inflammatory responses, while those from adaptive immune and non-immune compartments contribute more to immune regulation and vascular dysfunction. Together, these processes highlight the role of EVs in shaping the complex immune landscape of sepsis. Created with BioRender.com.

### 3.1. Immune Cell-Derived EVs: A Cell-Specific Perspective

#### 3.1.1. T Lymphocyte-Derived EVs

T lymphocytes are central regulators of adaptive immunity and have a critical influence on the development of sepsis-associated immunosuppression [36]. EVs released by T cells reflect their activation and functional state and contribute to the modulation of immune responses during systemic infection [60].

T-cell-derived EVs carry surface molecules such as MHC protein, co-stimulatory receptors, and immune checkpoint ligands, as well as regulatory microRNAs that influence lymphocyte activation and differentiation [60]. Through these components, they can modulate both innate and adaptive immune responses in a cell-contact-independent manner [60,66,67,68].

A key mechanism linking T cell-derived EVs to immune dysfunction in sepsis involves immune checkpoint signaling. Increased expression of programmed death-1 (PD-1) on T cells is a hallmark of sepsis-induced immunosuppression, and elevated levels of EV-associated PD-L1 have been associated with poor clinical outcomes [67]. EV-associated PD-L1 can engage PD-1 on CD4+ T cells, contributing to T-cell dysfunction and impaired immune responses [67].

In addition to checkpoint signaling, T cell-derived EVs can transfer regulatory microRNAs that further influence immune responses. Vesicles enriched in miR-150-5p and miR-181a have been associated with reduced T-cell proliferation and altered immune regulation, supporting their contribution in the maintenance of an immunosuppressive environment [66].

Experimental studies have also identified more direct effects of T cell-derived EVs on tissue injury. For instance, EV-associated diacylglycerol kinase kappa (DGKK) has been shown to promote alveolar epithelial cell apoptosis and inflammation through activation of the DAG/PKC/NOX4 pathway in models of sepsis-induced lung injury [68].

Overall, T cell-derived EVs appear to act primarily as mediators of immune suppression in sepsis, reflecting and potentially reinforcing T-cell dysfunction [36]. Compared with innate immune cell-derived EVs, their role is less extensively characterized, and further studies are needed to define their contribution to immune regulation and clinical outcomes. 

#### 3.1.2. Neutrophil-Derived EVs

Neutrophils represent another major source of EVs during acute inflammatory responses, which are key effectors of innate immunity in sepsis [36]. Neutrophil-derived EVs contribute to immune regulation through multiple mechanisms, including modulation of cytokine release, promotion of chemotaxis, and interaction with neutrophil extracellular trap (NET) formation [60,69].

Experimental evidence suggests that neutrophil-derived EVs can influence inflammatory signaling in recipient cells [60]. These vesicles have been shown to carry regulatory microRNAs, such as miR-223, which modulate macrophage activation and attenuate the expression of pro-inflammatory cytokines, including IL-6 and IL-1β [70]. Through these mechanisms, neutrophil-derived EVs may participate in the fine-tuning of inflammatory responses during systemic infection.

At the same time, neutrophil-derived EVs have been implicated in the propagation of tissue injury. Increased release of neutrophil-derived EVs has been associated with enhanced inflammatory cascades, endothelial activation, and the promotion of immunothrombosis, in part through interactions with platelets and NET formation [28]. These processes contribute to microvascular dysfunction and organ damage, which are hallmarks of sepsis. 

Compared with other immune cell populations, the role of neutrophil-derived EVs in sepsis is still less well characterized. Current evidence is largely derived from experimental models or extrapolated from studies on related inflammatory conditions [60]. Nevertheless, available data suggest that neutrophil-derived EVs act as context-dependent regulators of inflammation, capable of exerting both pro- and anti-inflammatory effects depending on the microenvironment.

#### 3.1.3. Macrophage-Derived EVs

Macrophages and monocytes represent major sources of EVs during the systemic inflammatory response and macrophage-derived EVs act as central amplifiers of innate immune signaling in sepsis [69]. EVs released from activated monocytes and macrophages carry an ample repertoire of bioactive molecules, including cytokines, chemokines, inflammasome components, and regulatory microRNAs, enabling them to influence both immune and endothelial cell functions [60,69].

Accumulating experimental evidence indicates that macrophage-derived EVs contribute to the propagation of inflammatory signals across tissues [60]. Their cargo frequently includes molecules associated with inflammasome activation and cytokine signaling, supporting the transfer of pro-inflammatory stimuli to recipient cells [60]. In particular, macrophage-derived EVs enriched in chemokines such as CXCL2 have been shown to promote neutrophil recruitment and activation via the CXCR2/PKC/NOX4 signaling pathway, thereby amplifying inflammatory responses [71]. 

These vesicles have also been implicated in the development of organ injury. In experimental models of sepsis, macrophage-derived EVs exacerbate cytokine release, including TNF-α, IL-1β, and IL-6, and contribute to acute lung injury and myocardial dysfunction [72,73]. Mechanistically, this effect has been linked to the transfer of inflammasome-related components, such as TXNIP-NLRP3 complexes, leading to the activation of IL-1β and IL-18 signaling pathways [73].

At the same time, macrophage-derived EVs can exert regulatory functions. Certain EV-associated microRNAs, including miR-146a-5p, have been shown to modulate Toll-like receptor signaling and attenuate excessive inflammatory responses in recipient cells, including macrophages and other innate immune cells [74]. 

These findings highlight the context-dependent functions of macrophage-derived EVs, which may either amplify or limit inflammation depending on the activation state of the parent cells and the surrounding microenvironment [69].

#### 3.1.4. Dendritic Cell-Derived EVs

Dendritic cells (DCs) are central regulators of adaptive immunity and are critical for orchestrating the transition from innate to adaptive immune responses during sepsis [36]. EVs released by DCs extend these functions by enabling the transfer of antigenic and immunoregulatory signals between cells [60].

DC-derived EVs carry major histocompatibility complex (MHC) molecules, co-stimulatory proteins, and immunomodulatory microRNAs, allowing them to influence T-cell activation, differentiation, and immune polarization [60]. Through these mechanisms, DC-derived vesicles can modulate adaptive immune responses even in the absence of direct cell-to-cell contact.

Experimental studies have shown that DC-derived EVs can transfer antigenic material to other antigen-presenting cells and contribute to the amplification and dissemination of immune responses. This capacity supports the concept that EVs act as cell-free extensions of DC function, enabling coordinated immune signaling across tissues.

The ability of DC-derived EVs to mediate long-range communication is further highlighted by their capacity to cross biological barriers, including the blood–brain barrier in engineered systems [75]. Although available data are merely experimental, they suggest the potential contribution of DC-derived EVs to the coordination of adaptive immune responses and the integration of immune signaling during systemic infection, even at a distance from their site of origin.

### 3.2. Non-Immune Cell-Derived EVs

In addition to the central contribution of EVs released by immune cells to shaping inflammatory signaling during sepsis, the circulating vesicle pool also includes substantial contributions from non-immune cellular compartments [21,44,69]. Endothelial cells, platelets, and mesenchymal stem cells release EVs that reflect tissue stress and vascular activation as well as organ dysfunction [69]. These vesicles provide an additional layer of intercellular communication linking immune responses with coagulation, endothelial dysfunction, and tissue injury [69].

#### 3.2.1. Endothelial Cell-Derived EVs

Endothelial cells (ECs) actively coordinate immune responses during sepsis by regulating leukocyte trafficking, vascular inflammation, and interactions between circulating immune cells and tissues [36,69]. EC-derived EVs reflect this activated state and extend endothelial–immune communication beyond the vascular compartment. These vesicles carry adhesion molecules, inflammatory mediators, and regulatory microRNAs that influence leukocyte recruitment, monocyte differentiation, and inflammatory signaling [36,69]. Through these mechanisms, EC-derived EVs actively participate in shaping immune cell trafficking and the spatial organization of inflammation [76,77].

Experimental studies suggest that endothelial EVs modulate immune–vascular interactions by regulating pathways involved in leukocyte adhesion and migration, including VCAM1-ITGA4 signaling [76,77]. In addition, EV-associated microRNAs, such as miR-126, contribute to the regulation of inflammatory signaling and endothelial–immune crosstalk, influencing cytokine production and vascular responses [76,77].

Overall, EC-derived EVs function as integrators of immune and vascular responses, linking endothelial dysfunction to immune dysregulation during sepsis.

#### 3.2.2. Platelet-Derived EVs

Platelets are increasingly recognized as active participants in innate immunity, contributing not only to hemostasis, but also to inflammatory and host defense responses during sepsis [78,79,80]. Platelet-derived EVs are among the most abundant vesicle populations in circulation and are key mediators linking immune activation to coagulation pathways [79,80].

Rather than acting as classical immunomodulators, platelet-derived EVs contribute to immune dysregulation primarily through the promotion of immunothrombosis [28]. By transporting tissue factors and interacting with NETs, these vesicles amplify thromboinflammatory processes that are integral to the host response to infection [79,80,81,82]. This interplay between coagulation and innate immunity contributes to microvascular injury, impaired tissue perfusion, and organ dysfunction, which are hallmarks of sepsis [80].

Platelet-derived EVs also influence immune cell behavior through interactions with neutrophils, monocytes and endothelial cells, promoting inflammatory signaling and leukocyte recruitment [80]. Through these mechanisms, they act as key mediators at the interface between inflammation and coagulation, influencing the spatial and functional organization of the immune response [60,80].

Although their direct involvement in adaptive immune regulation is not fully characterized, platelet-derived EVs are increasingly recognized as critical drivers of thromboinflammatory responses that contribute to immune dysregulation in sepsis [28].

#### 3.2.3. Mesenchymal Stem Cell-Derived EVs

Mesenchymal stem cell (MSC)-derived EVs have attracted considerable attention in sepsis due to their immunomodulatory and therapeutic potential [69,83,84,85,86]. Unlike EVs derived from immune or vascular cells, which often propagate inflammatory signaling, MSC-EVs are predominantly associated with the attenuation of immune responses and the restoration of immune homeostasis. MSC-derived EVs recapitulate many of the immunosuppressive properties of their parent cells, including the ability to inhibit T-cell activation and proliferation and modulate macrophage function. These EVs carry bioactive molecules, including regulatory microRNA and proteins, that influence key inflammatory signaling pathways and cellular activation states [86,87].

A central mechanism underlying their effects involves the modulation of macrophage polarization. In preclinical models of sepsis, MSC-derived EVs promote a shift from pro-inflammatory M1 macrophages toward an anti-inflammatory M2 phenotype, thereby contributing to the resolution of inflammation. This process is mediated, at least in part, by EV-associated microRNAs, such as miR-21 and miR-146a, which regulate pathways including NF-κB and TRAF6 [86,88,89].

Additional experimental data show that MSC-derived EVs can reduce oxidative stress, inhibit inflammatory signaling cascades such as MAPK/NF-κB, and improve endothelial and epithelial barrier function, leading to decreased vascular permeability and reduced tissue injury in experimental models of sepsis [86,90].

Taken together, MSC-derived EVs represent a distinct class of vesicles with predominantly anti-inflammatory and tissue-protective properties. Their ability to modulate immune responses and restore immune balance in experimental models highlights their potential not only as therapeutic agents, but also as models of endogenous mechanisms that counteract immune dysregulation in sepsis. However, several issues currently limit their translation into clinical applications, which include an incomplete understanding of EV biodistribution and *in vivo* kinetics, potential off-target effects, and uncertainties regarding optimal dosing strategies. In addition, large-scale production, standardization of EV isolation, and quality control remain significant obstacles for clinical-grade manufacturing [82]. Finally, safety considerations, including long-term effects and immunogenicity, also require further investigation. These limitations underscore the need for rigorous preclinical and clinical studies in order to define the feasibility and safety of MSC-derived EV-based therapies in sepsis.

A summary of the main EV populations and their role in sepsis-associated immune dysregulation is provided in Table 1.

### 3.3. Integrative Perspective Across EV Sources

Across different cellular sources, EVs display both convergent and divergent functions in sepsis-associated immune dysregulation. A shared feature is their ability to mediate intercellular communication and modulate key pathways involved in inflammation, coagulation, and immune suppression [26,28,29,91]. However, their functional impact greatly varies depending on their cellular origin [26,29]. EVs derived from innate immune cells, such as macrophages and neutrophils, predominantly amplify and propagate inflammatory responses, whereas those from adaptive immune cells are more closely linked to the regulation of immune activation and the development of immunosuppressive states [60,71]. In parallel, vesicles released by non-immune cells, including endothelial cells and platelets, primarily connect immune activation with vascular dysfunction and immunothrombosis [26,28,61,77]. This functional heterogeneity underscores the importance of considering EV origin when interpreting their biological role and supports the view that EV populations collectively contribute to the dynamic immune landscape of sepsis.

## 4. Closing the Gap: Challenges and Future Directions

The concept of sepsis immune dysregulation has evolved beyond the traditional dichotomy of hyperinflammation versus immunosuppression, moving toward a more complex framework encompassing immune tolerance, metabolic adaptation, and dynamic immune reprogramming [21,36,40,50]. Despite recent advances, immunomodulatory therapy in sepsis continues to be largely empirical, reflecting the persistent gap between mechanistic understanding and clinical implementation [16,35,50]. The central challenge is no longer conceptual, but operational: how to translate heterogeneous and time-dependent immune states into actionable therapeutic strategies.

EVs offer a potential bridge and may represent an effective instrument to close the gap between biological complexity and clinical decision-making. By encapsulating coordinated molecular signals that reflect immune activation, suppression and metabolic rewiring, EVs provide a dynamic and cell-specific readout of the host response [26,28,60]. Unlike conventional biomarkers, which capture isolated aspects of inflammation, EV profiles may enable the monitoring of immune trajectories over time, supporting a more adaptive and individualized approach to therapy [52,60,69].

Recent technological developments are beginning to support this paradigm. High-resolution approaches, including single-vesicle analysis, multimodal omics integration, and computational modeling, allow increasingly refined characterization of EV populations. When combined with longitudinal sampling and machine learning-based analysis, these strategies may facilitate the identification of reproducible immune signatures and the stratification of patients into biologically defined subgroups [24,26,60,77]. Building on these advances, an important next step is to move EV-based approaches beyond static measurements toward dynamic and clinically actionable frameworks. Longitudinal EV profiling would enable the tracking of immune trajectories over time, offering insights not captured by single-time-point assessments. In parallel, the identification of quantitative thresholds associated with distinct immune states could support patient stratification and risk assessment. Integration of EV-derived signatures with clinical parameters and other biomarkers may further enhance their utility, informing treatment selection and monitoring of therapeutic responses. While these applications remain largely conceptual and require further validation, they underscore the potential of EVs to contribute to real-time clinical decision-making in sepsis.

Still, substantial challenges persist. A major limitation in EV research lies in pre-analytical and methodological variability. Factors such as sample type, collection procedures, processing, storage conditions, and isolation techniques can substantially affect EV yield, composition, and downstream analysis, contributing to poor reproducibility across studies and hindering standardization [92]. In addition, several issues constrain clinical translation, including the lack of robust and reproducible isolation and characterization methods, scalability limitations, platform variability, and the absence of validated reference frameworks [93,94,95]. Regulatory requirements, manufacturing complexity, cost, and turnaround time further complicate integration into routine clinical practice [92]. Emerging approaches, including automated isolation platforms, microfluidic technologies, and the development of consensus protocols, may help mitigate some of these limitations and facilitate clinical implementation [52,96]. Addressing these issues will be essential to advance the EV-based approach from experimental settings to clinically applicable tools. Moreover, reference frameworks linking EV profiles to defined immune phenotypes are still lacking, and most available data are derived from preclinical studies or small patient cohorts. In addition, prospective trials incorporating EV-based stratification into therapeutic decision-making are largely absent, limiting current clinical applicability.

A key unresolved aspect of EV-based approaches concerns the optimal timing and frequency of measurement. Most studies rely on single-time-point analysis, which may fail to capture the dynamic nature of immune responses in sepsis. Given the rapidly evolving immune landscape, longitudinal sampling strategies may provide more informative insights into patient-specific trajectories. Repeated EV profiling at defined clinical stages—such as early infection, peak disease severity, and recovery—could improve the assessment of immune dynamics and support patient stratification. However, standardized protocols for sampling, frequency, and analytical workflows are currently lacking and represent an important area for future research. 

Therefore, at the current stage of our knowledge, EV-based approaches can be considered by clinicians as the only possible emerging tool rather than an immediate alternative to established biomarkers. Their future impact will depend on their ability to complement existing strategies and provide clinically applicable information within realistic timeframes. Beyond their diagnostic applications, EVs are also being explored as therapeutic platforms. Their capacity for targeted cargo delivery and intercellular communication raises the possibility of modulating immune responses in a controlled and cell-specific manner [60]. Approaches based on engineered EVs or on the modulation of endogenous vesicle release may, in principle, enable the attenuation of maladaptive inflammation, the reversal of immunosuppression, or the reprogramming of immune–metabolic pathways associated with disease tolerance [97]. At present, these strategies are confined to experimental settings and require further validation in clinically relevant applications.

A major limitation of the current evidence is, indeed, that most findings are derived from preclinical models or small, heterogeneous patient cohorts, with limited validation in large, prospective clinical studies. In particular, robust clinical trial data specifically evaluating EV-based approaches in sepsis are still lacking. As a result, the clinical applicability of EV-based approaches remains to be determined. Well-designed, large-scale prospective studies will be essential to validate the generalizability of the current knowledge, define clinically relevant thresholds, and assess their utility for patient stratification and therapeutic decision-making.

The central question for the coming decade is not simply whether EVs participate in sepsis pathophysiology, but whether they can serve as dynamic biomarkers and therapeutic vectors capable of bridging immune complexity with therapeutic precision. Addressing this question will determine whether EV research remains descriptive and barely informative or if it will become truly transformative for sepsis care. 

## Figures and Tables

**Table 1:** Cell-specific extracellular vesicles in sepsis-associated immune dysregulation: from mechanistic insights to translational challenges.

Cellular Source	Representative EV Cargo	Immune Function	Role in Sepsis Immune Dysregulation	Clinical Relevance/Translational Potential	Level of Evidence	Key Limitations
**Macrophages/Monocytes**	Cytokines, CXCL2, NLRP3, inflammasome components, miR-146a	Amplification and modulation of innate immune responses	Propagation of inflammation; transfer of inflammasome signals; context-dependent regulation of immune activation	Potential markers of inflammatory phenotypes, targets for modulation of innate immune signaling	Preclinical > Translational	Limited clinical validation; model-dependent findings
**Neutrophils**	miR-223, NET-associated components	Regulation of inflammatory responses; interaction with innate immune cells	Fine-tuning of inflammation; contribution to immunothrombosis and tissue injury	Indicators of disease severity and thromboinflammatory activity	Preclinical	Sparse human data; heterogeneity across models
**Dendritic cells**	MHC molecules, co-stimulatory proteins, regulatory miRNAs	Antigen presentation and adaptive immune activation	Coordination of adaptive immune responses; intercellular transfer of antigenic signals	Limited evidence; potential role in immune monitoring	Preclinical (limited)	Very limited data; poorly defined in sepsis
**T lymphocytes**	PD-L1, miR-150-5p, miR-181a	Regulation of adaptive immunity; immune checkpoint signaling	Contribution to immunosuppression and T-cell dysfunction; reinforcement of exhausted phenotypes	Biomarkers of immune suppression; potential targets for checkpoint modulation	Translational/early clinical	Limited cohort size; lack of validation
**Endothelial cells**	Adhesion molecules (ICAM-1, VCAM-1), miR-126	Immune–vascular crosstalk; regulation of leukocyte trafficking	Promotion of leukocyte adhesion, endothelial activation, and vascular inflammation; contribution to organ dysfunction	Biomarkers of endothelial activation and ARDS; targets for vascular–immune modulation	Preclinical > Translational	Limited human validation; context variability
**Platelets**	Tissue factor, procoagulant molecules, miRNAs	Immunothrombosis and innate immune activation	Amplification of thromboinflammatory responses; interaction with NETs; microvascular injury	Indicators of coagulopathy; potential targets for modulating thromboinflammation	Translational	Quantitative contribution unclear; limited mechanistic clinical data
**Mesenchymal stem cells (MSCs)**	miR-21, miR-146a, anti-inflammatory mediators	Immunomodulation and restoration of immune homeostasis	Promotion of anti-inflammatory macrophage polarization; attenuation of inflammatory signaling	Promising therapeutic platform; EV-based immunotherapeutic strategies	Preclinical	Lack of clinical trials; safety and scalability concerns

EVs, extracellular vesicles; NETs, neutrophil extracellular traps; ARDS, acute respiratory distress syndrome.

## Data Availability

No new data were created or analyzed in this study.
